# Predictors of Mortality in Surgical Patients Admitted to a Tertiary Intensive Care Unit

**DOI:** 10.3390/jcm14186369

**Published:** 2025-09-09

**Authors:** Tolga Girgin, Volkan Sayur, Erkan Güler, Can Uç, Berk Göktepe, Sinan Ersin, Mehmet Uyar, Taylan Özgür Sezer

**Affiliations:** 1 Department of Surgery, Ege University School of Medicine, İzmir 35100, Turkey; tolgagirgin0@gmail.com (T.G.); sayurvolkan@gmail.com (V.S.); drcanuc@gmail.com (C.U.); berkgoktepe@gmail.com (B.G.); sersin@gmail.com (S.E.);; 2 Department of Surgery, Mersin University School of Medicine, Mersin 33110, Turkey; 3 Department of Anesthesia, Ege University School of Medicine, İzmir 35100, Turkey

**Keywords:** inflammatory biomarkers, mortality predictors, prognostic scoring systems, surgical critical care

## Abstract

**Background**: Intensive Care Units (ICUs) provide critical support for patients after major surgery or acute abdominal conditions. Despite medical advances, mortality remains high in surgical ICU patients. This study aimed to identify clinical and biochemical predictors of mortality in surgical patients admitted to a tertiary ICU. **Methods**: We conducted a retrospective case–control study on 231 adult general surgery patients admitted to a tertiary anesthesia ICU between January 2018 and December 2023. Patients under 18 years or who underwent solid organ transplantation were excluded. Data collected included demographic, clinical, and laboratory parameters such as the Glasgow Coma Scale (GCS), Acute Physiology and Chronic Health Evaluation II (APACHE II), Sequential Organ Failure Assessment (SOFA), Hemoglobin-Albumin-Lymphocyte-Platelet (HALP) score, neutrophil-to-lymphocyte ratio (NLR), and C-reactive protein (CRP)/albumin ratio. Patients were divided into mortality and survival groups, with subgroup analyses performed for malignancy, sepsis, and trauma. Receiver operating characteristic (ROC) curve and Cox regression analyses were used to identify mortality predictors. **Results**: The ICU mortality rate was 64.9%. Significant predictors included age ≥ 58 years (odds ratio [OR] 4.56), body mass index (BMI) > 30 kg/m^2^ (OR 7.62), mean arterial pressure < 70 mmHg (OR 1.66), serum albumin < 21.3 g/L (OR 1.5), APACHE II > 18.5 (OR 2.42), and SOFA > 9.5 (OR 2.68). Mortality was also associated with lower GCS scores, prolonged mechanical ventilation, and inotropic support. The CRP/albumin ratio was significantly elevated in the mortality group (*p* = 0.024). Other inflammatory markers showed no significant differences. Predictive factors varied among subgroups. **Conclusions**: Older age, obesity, hypotension, hypoalbuminemia, and high severity scores independently predict mortality in surgical ICU patients. Early risk identification may enhance management and improve outcomes in this population.

## 1. Introduction

Intensive Care Units (ICUs) are specialized units where advanced life-support systems are utilized to monitor and treat patients with critical health conditions [1]. After major and specialized surgeries in general surgery or due to conditions such as acute mesenteric ischemia, intra-abdominal sepsis, severe pancreatitis, or trauma, patients often require monitoring in tertiary-level intensive care units. Therefore, general surgery and ICU services work closely together. Worldwide, approximately 310 million surgical procedures are performed annually. Around 7 million patients experience serious perioperative morbidity, and about 0.5% of surgeries result in mortality [2,3]. Advances in surgical techniques and postoperative care have allowed more complex surgeries to be performed, increasing the frequency of interventions in patients with severe health problems [4,5].

Septic shock and cardiogenic shock have been reported as leading causes of mortality in surgical ICU patients. Scoring systems such as APACHE, SAPS, and SOFA have been developed to predict mortality. Despite certain limitations, these scoring systems are widely used in clinical practice. Beyond scoring systems, factors such as a low Glasgow Coma Scale (GCS) score (<9), prolonged mechanical ventilation, inotropic support, lactic acidosis, massive transfusion, the presence of comorbidities, hypoalbuminemia, emergency surgical interventions, and advanced age have been reported to be associated with mortality in surgical ICUs [6,7]. Literature data specific to tertiary ICUs mainly investigate mortality-related factors in cardiovascular and neurosurgical patients. Reported mortality rates for ICU patients range between 20.5% and 43%, with the most common causes of death being sepsis, cardiopulmonary arrest, pneumonia, and malignant arrhythmias. Factors affecting mortality in general surgery-related pathologies requiring intensive care have not been sufficiently addressed.

However, much of the current literature focuses on cardiovascular and neurosurgical ICU populations, while evidence addressing predictors of mortality in general surgery patients requiring tertiary ICU care remains limited. This gap is particularly important, as these patients frequently present with complex abdominal pathologies, higher comorbidity burdens, and significant postoperative complications. The present study aims to address this gap by identifying clinical and biochemical predictors of mortality specific to general surgery patients in a tertiary ICU setting, thereby providing data that may guide risk stratification and optimize patient management.

## 2. Methods

### 2.1. Study Design and Ethical Approval

This retrospective case-control study was approved by the Ege University Medical Research Ethics Committee on 23 May 2024 (Approval No: 24-5.1T/25).

This retrospective study was approved by the institutional ethics committee. Due to the retrospective nature of the study and the critical condition of many patients (including those who were intubated), the need to obtain informed consent was waived by the ethics committee.

### 2.2. Study Population and Inclusion Criteria

Patients aged 18 years and older with a history of general surgery-related pathology who were transferred from the General Surgery Clinic to the tertiary Anesthesia Intensive Care Unit between January 2018 and December 2023 were included. Patients who had undergone solid organ transplantation (liver or kidney) or were under 18 years old were excluded due to differing clinical courses and prognostic outcomes. Additional exclusion criteria included incomplete medical records and admission to the ICU from non-surgical clinics. At ICU admission, the type of respiratory support was documented for all patients, including spontaneous breathing, oxygen mask, orotracheal intubation, or tracheostomy. Mechanical ventilation and duration of intubation were also recorded, as these parameters were later analyzed in relation to mortality.

## 3. Data Collection

This was a retrospective study, and all eligible patients admitted consecutively to the ICU during the study period were included. Data were obtained from electronic hospital information systems and supplemented by physical patient files when necessary. Demographic data, clinical parameters, laboratory values, and severity scores (SOFA, APACHE II, GCS) were extracted using a standardized case form and transferred into an electronic spreadsheet for analysis.

Patients were divided into two groups based on mortality during ICU stay: the mortality group and the non-mortality group (discharged patients). Clinical, laboratory, and scoring parameters were compared between these groups. Demographic and descriptive parameters including age, sex, body mass index (BMI; weight in kg/height in m^2^), comorbidities, diagnosis, vital signs (blood pressure, pulse, and urine output), respiratory support type (spontaneous breathing, oxygen mask, orotracheal intubation, and tracheostomy), Glasgow Coma Scale (GCS), APACHE II, SOFA, HALP scores, neutrophil/lymphocyte ratio, neutrophil/platelet ratio, CRP/albumin ratio, platelet/lymphocyte ratio, need for inotropes, hemodialysis, transfusion requirements, and discharge status (alive or deceased) were evaluated. Subgroup analyses based on etiology (malignancy, sepsis, and trauma) were performed to assess factors affecting mortality. For severity assessment, validated prognostic scoring systems were used. The APACHE II score evaluates disease severity and short-term mortality risk based on 12 routine physiological measurements (temperature, mean arterial pressure, heart rate, respiratory rate, oxygenation, arterial pH, serum sodium, potassium, creatinine, hematocrit, white blood cell count, and Glasgow Coma Scale), along with age and chronic health status. The SOFA score was calculated to assess organ dysfunction, including six systems: respiratory (PaO_2_/FiO_2_ ratio), coagulation (platelet count), liver (bilirubin level), cardiovascular (blood pressure and the need for vasopressors), the central nervous system (Glasgow Coma Scale), and renal function (creatinine or urine output). These validated scoring systems are widely used to stratify critically ill patients and predict ICU outcomes.

## 4. Statistical Analysis

Data were analyzed using version 29.0 (SPSS Inc., Chicago, IL, USA). Descriptive statistics were presented as numbers (*n*) and percentages (%) for categorical variables, and mean ± standard deviation for normally distributed continuous variables. A 95% confidence interval was used, with *p*-values < 0.05 considered statistically significant. Normality of continuous data was assessed using Kolmogorov–Smirnov and Shapiro–Wilk tests. Normally distributed continuous variables were compared between groups using Student’s *t*-test or One-Way ANOVA, while non-normally distributed data were analyzed using Mann–Whitney U and Kruskal–Wallis tests. Categorical variables were compared with the Chi-square test; Spearman’s correlation was applied when appropriate. Survival analysis was conducted using Kaplan–Meier methods. For parameters showing significant differences between groups, cut-off values were identified via ROC curve analysis. Parameters with area under the curve (AUC) greater than 65% and significant *p*-values were further evaluated using the Youden index to determine optimal cut-off points. Patients were then dichotomized based on these cut-offs. Cox regression analysis was performed to evaluate the combined effect of these parameters on survival. Receiver operating characteristic (ROC) curve analysis was used to evaluate the predictive performance of continuous variables (e.g., age, BMI, serum albumin, APACHE II, SOFA, and GCS scores) that were significantly different between groups. For each variable, the area under the curve (AUC) was calculated. Parameters with an AUC greater than 0.65 and statistically significant discrimination were selected for further evaluation. The optimal cut-off point for each parameter was determined using the Youden Index (sensitivity + specificity − 1), which maximizes the balance between sensitivity and specificity. Patients were then categorized into binary groups according to these cut-offs, which were subsequently included in Cox regression analysis to identify independent predictors of mortality.

## 5. Results

Between January 2018 and December 2023, 259 patients transferred from the General Surgery Clinic to the Anesthesia Intensive Care Unit were screened. Sixteen patients with liver transplants, two with kidney transplants, and ten patients with missing data were excluded. A total of 231 patients were included, of whom 90 (39%) were female and 141 (61%) were male. The mean age was 59.49 ± 18.43 years, and the mean BMI was 28.85 ± 6.04. At ICU admission, 74.02% of patients had at least one comorbidity.

The primary diagnoses included gastrointestinal perforation in 57 patients (24.7%), mesenteric vascular disease in 19 (8.2%), mechanical bowel obstruction in 35 (15.2%), acute biliopancreatic infections (pancreatitis, cholecystitis, and cholangitis) in 18 (7.8%), trauma in 44 (19%), necrotizing fasciitis in 5 (2.2%), gastrointestinal bleeding in 11 (4.8%), and elective cancer surgery requiring postoperative ICU care in 42 (18.2%). Among these patients, 150 (64.9%) died during their ICU stay, while 81 (35.1%) were discharged alive. The ICU mortality rate for general surgery patients was 64.9%. The mean length of ICU stay was 11.38 ± 16.59 days. No significant differences were found between mortality and non-mortality groups in inflammatory indices, including the neutrophil/lymphocyte ratio, platelet/lymphocyte ratio, neutrophil/platelet ratio, and HALP score (*p* = 0.064, *p* = 0.947, *p* = 0.129, *p* = 0.072, respectively). However, the CRP/albumin ratio was significantly higher in the mortality group (*p* = 0.024). Decreased GCS scores at ICU admission were associated with mortality, while higher SOFA and APACHE II scores were significantly higher in the mortality group (*p* < 0.001) (Table 1).

### 5.1. Malignancy Group

Among 231 patients, 44 had a diagnosis of malignancy. Of these, 30 (68.18%) died, and 14 (31.81%) were discharged alive. Significant differences between mortality groups were found for the APACHE II, HALP score, CRP/albumin ratio, neutrophil/lymphocyte ratio (NLR), and thrombocyte/lymphocyte ratio (TLR) (*p* = 0.002, *p* = 0.001, *p* = 0.047, *p* = 0.008, *p* = 0.003, respectively) (Table 2).

### 5.2. Sepsis Group

Sepsis was present in 134 patients (58.01%) due to diagnoses such as gastrointestinal perforation, mesenteric vascular disease, mechanical bowel obstruction, biliopancreatic infections, or necrotizing fasciitis. Among these, the mortality group had a higher mean BMI (30.33 ± 6.88) compared to the non-mortality group (27.13 ± 5.54) (*p* < 0.05). Significant differences were observed between groups for APACHE II, SOFA, and thrombocyte/lymphocyte ratio (*p* < 0.001, *p* = 0.001, *p* = 0.036, respectively). Other parameters showed no significant differences (Table 3).

### 5.3. Trauma Group

There were 44 trauma patients requiring ICU care, of whom 14 (31.81%) died and 30 (68.18%) were discharged alive. The mean BMI was 28.45 ± 6.48 in the mortality group and 26.17 ± 2.58 in the non-mortality group (*p* = 0.267). Significant differences between groups were noted for APACHE II and SOFA scores (*p* < 0.001), but not for other parameters (Table 4). Regarding blood transfusions, 13 patients (29.54%) did not require transfusion, while 31 (70.4%) received transfusions; among these, 10 (22.72%) had massive transfusions. No significant difference was found between groups regarding transfusion needs (*p* = 0.130).

### 5.4. Factors Affecting Mortality

Sex was not found to be a significant factor affecting mortality. Mortality was significantly higher in patients who were intubated during ICU monitoring compared to those on oxygen mask or room air (*p* < 0.001). Patients with impaired consciousness (closed or confused) had higher mortality compared to cooperative and oriented patients (*p* < 0.001). The need for inotropic support, higher inotropic dose, and hypotension at ICU admission were significantly associated with increased mortality (*p* < 0.05).

**Figure 1:**
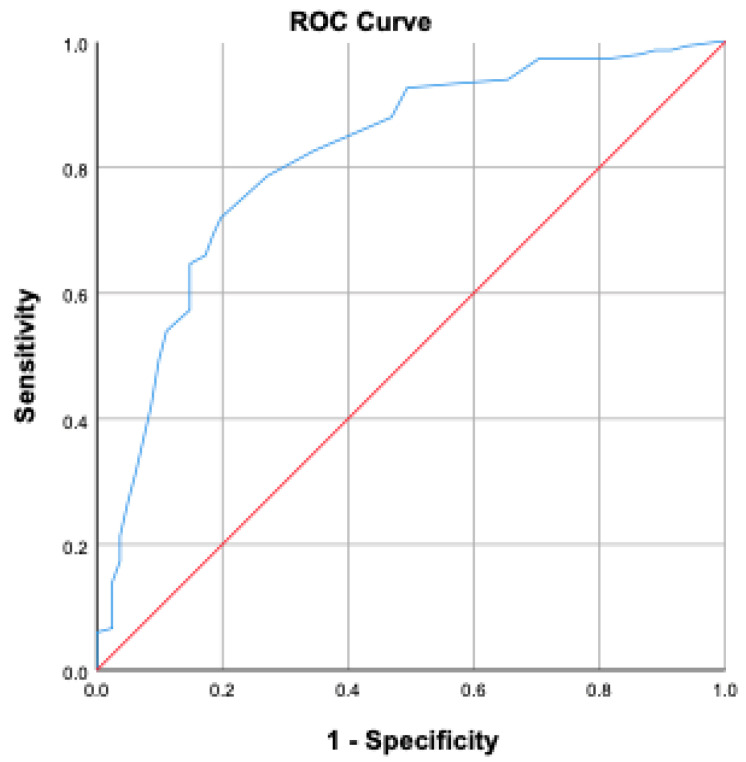
Evaluation of APACHE II score for mortality prediction using ROC curve.

**Figure 2:**
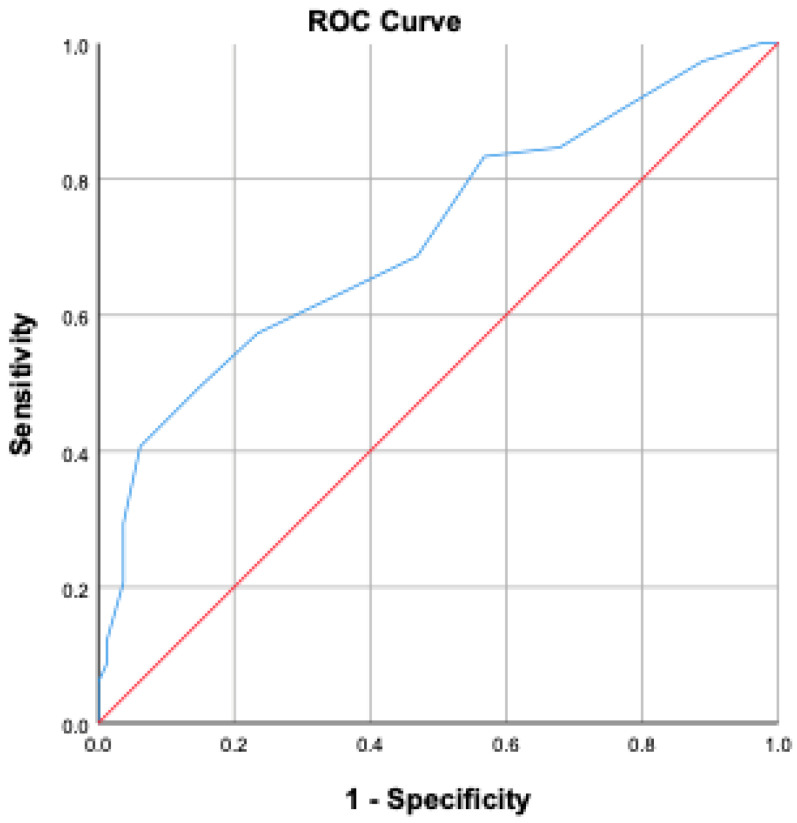
ROC curve demonstrating the predictive value of SOFA score for mortality.

BMI was significantly higher in the mortality group compared to survivors (*p* < 0.001). Oliguria and the need for hemodialysis during ICU stay were significantly higher in the mortality group (*p* < 0.001). No significant differences were found regarding blood transfusion or blood glucose levels (*p* = 0.067 and *p* = 0.487, respectively). The average length of ICU stay was 11.65 ± 17.9 days in the mortality group and 10.87 ± 13.92 days in the non-mortality group (*p* = 0.379). However, intubation duration was significantly longer in the mortality group (11.26 ± 16.97 days) compared to the non-mortality group (5.93 ± 12.93 days) (*p* < 0.001). Age ≥ 58 years increased mortality risk by 4.56 times; BMI > 30 increased it by 7.62 times; mean arterial pressure < 70 mmHg increased it by 1.66 times; serum albumin < 21.3 g/L increased it by 1.5 times; APACHE II score > 18.5 increased it by 2.42 times; and SOFA score > 9.5 increased it by 2.68 times. ROC analyses are presented in Figure 1 and Figure 2.

Figure 1 and Figure 2 present ROC curve analyses derived from our study cohort, illustrating the discriminatory ability of APACHE II and SOFA scores, respectively, in predicting ICU mortality among surgical patients.

## 6. Discussion

This retrospective study evaluated surgical ICU patients over a five-year period, focusing on mortality rates and associated risk factors. Our findings revealed a high ICU mortality rate of 64.9%, which is influenced by the exclusive inclusion of general surgery patients with complex clinical profiles. Key factors such as advanced age, obesity, inflammatory markers, including NLR and TLR in malignancy patients, and APACHE II and SOFA scores were significantly associated with mortality. Additionally, the CRP/albumin ratio demonstrated prognostic value in predicting outcomes, especially among patients with malignancies. These results emphasize the multifactorial nature of mortality risk in surgical ICU patients and highlight important predictive parameters that can guide clinical management.

Advanced age, comorbidities, and disease severity represent independent risk factors for both short- and long-term mortality. Mortality rates increase significantly with age, especially in patients over 80 years old [8,9]. While advanced age alone does not prevent successful treatment, realistic expectations regarding outcomes require considering the overall health status and comorbidities of elderly patients. Obesity affects multiple systems and alters the expected metabolic response to conditions such as sepsis, trauma, and ischemia. Additional strain on cardiovascular and respiratory functions decreases the survival probability of obese patients during critical illness. Drug distribution and metabolism also differ in obese individuals, presenting further therapeutic challenges. Consequently, increased complications associated with obesity appear consistently across multiple studies [10,11,12]. Advanced age and BMI were identified as significant risk factors for mortality in our study (*p* < 0.001).

The neutrophil-to-lymphocyte ratio (NLR) has been utilized since the early 2000s as a parameter in endotoxemia, SIRS, and sepsis [13]. The platelet-to-lymphocyte ratio (PLR) serves as a prognostic marker in inflammation, sepsis, trauma, pneumonia, colorectal cancer, and cardiac surgery [14,15,16]. The neutrophil-to-platelet ratio (NPR) reflects an index of acute inflammatory response superimposed on chronic inflammation and acts as a prognostic biomarker in myocardial infarction and ulcerative colitis [17,18].

Across the entire cohort, these parameters did not exhibit statistically significant differences. Due to the heterogeneous patient population, subgroup analyses were conducted. In the malignancy subgroup, NLR and PLR demonstrated significance. A systematic meta-analysis indicates these ratios can predict outcomes in solid tumors and correlate with poor survival [19]. Within this subgroup, average NLR was 12.64 ± 8.34 in non-survivors compared to 6.5 ± 4.15 in survivors, consistent with other studies [20]. NLR and PLR ratios thus offer prognostic insights for surgical ICU patients with malignancies. In the sepsis subgroup, only PLR showed statistical significance; although differences in NLR appeared, these were not statistically significant, possibly due to the inclusion of leukopenic and neutropenic patients affecting correlation. NPR did not show significance in any subgroup, suggesting the need for further research with alternative study designs.

The HALP score is an immunonutritional parameter developed for predicting survival, disease-free survival, and mortality risk in malignancy patients [21]. It serves as a prognostic marker across several cancer types, including gastric, colorectal, bladder, prostate, kidney, esophageal, pharyngeal, lung, breast, and cervical cancers [22,23]. Despite its theoretical prognostic potential, it has not yet been widely integrated into clinical practice to guide nutritional interventions. Our study population included internal medicine, surgical, and emergency admissions; thus, heterogeneity likely contributed to a lack of significant difference in the overall cohort. However, HALP proved useful in predicting mortality within the malignancy subgroup. Its predictive capacity in acute inflammatory conditions such as sepsis and septic shock appears limited. Larger studies are required to validate these findings due to small sample sizes in subgroups.

The CRP-to-albumin ratio combines nutritional and inflammatory status and has been extensively examined as an independent prognostic marker in sepsis, infections, malignancies, and other diseases. However, few studies have specifically evaluated its prognostic value in critically ill surgical ICU patients [24,25]. The CRP/albumin ratio was significantly higher among non-survivors and aligned with the literature in the malignancy subgroup. In the sepsis subgroup, the expected significance was not observed, likely due to the limited sample size and borderline values. Previous studies have suggested its independent predictive value for 28-day mortality in critical ICU patients [26]. APACHE II and SOFA scores are widely recognized and accepted tools for mortality prediction in surgical ICU patients [27]. Both scores were significantly higher among non-survivors. Subgroup analyses confirmed higher scores in the mortality groups, although the SOFA score did not differ significantly among malignancy patients. A notable limitation of APACHE II is the absence of variables related to hemodynamic support and mechanical ventilation, which suggests incorporating these parameters could enhance predictive accuracy. Some nonsignificant results in subgroups may be attributed to small sample sizes and heterogeneous distributions. Multivariate analyses support the use of both scores as independent predictors of mortality. From a clinical perspective, our results suggest that early recognition of high-risk features—such as advanced age, obesity, hypoalbuminemia, and elevated APACHE II and SOFA scores—can support proactive decision-making in the ICU. Identifying such patients upon admission may justify prioritizing closer hemodynamic monitoring, timely initiation of mechanical ventilation or renal replacement therapy, and earlier involvement of multidisciplinary teams. Furthermore, the prognostic role of the CRP/albumin ratio, particularly in malignancy patients, underlines the importance of combining inflammatory and nutritional assessment in risk stratification. Incorporating these markers into routine practice could improve individualized care strategies, optimize ICU resource utilization, and potentially improve survival outcomes in surgical ICU populations.

This study has several limitations. First, it was conducted in a single tertiary center with a retrospective design, which restricts the generalizability of the findings. Second, although the overall sample size was moderate, subgroup analyses were limited by smaller numbers, which may have reduced statistical power. Third, certain potential confounders, such as detailed nutritional status, perioperative care variations, and socioeconomic factors, could not be evaluated due to incomplete data in retrospective records. Fourth, the cut-off values identified for the CRP/albumin ratio, APACHE II, and SOFA scores were derived from our own cohort and require external validation before clinical implementation. Finally, owing to the observational design, our study can only identify associations and does not allow causal relationships to be established. Despite these limitations, the study provides valuable insights into predictors of mortality in surgical ICU patients and highlights directions for future multicenter prospective research.

## 7. Conclusions

The parameters examined in this study demonstrate significant predictive value in determining mortality rates among surgical intensive care patients and optimizing ICU resource utilization. Our findings highlight the complexity and high risk of mortality and morbidity in general surgical ICU patients, emphasizing the need for meticulous care. Age, obesity, comorbidities, hemodynamic status, and renal parameters all significantly impact mortality outcomes. Additionally, established scoring systems such as APACHE II and SOFA remain effective tools for mortality prediction. Furthermore, the CRP/albumin ratio, reflecting both inflammatory response and nutritional status, showed significant prognostic importance, particularly within the malignancy subgroup. Elevated CRP/albumin values correlated with higher mortality, suggesting its potential as an independent predictor and a useful parameter for risk stratification in critically ill surgical patients.

## Figures and Tables

**Table 1:** Comparison of inflammatory biomarkers and clinical severity scores between survivors and non-survivors.

Parameter	Non-Survivors (*n* = 150) Mean ± SD	Survivors (*n* = 81) Mean ± SD	*p*-Value
Inflammatory Markers			
Neutrophil/Lymphocyte Ratio (NLR)	8.67 ± 6.46	6.93 ± 7.77	0.064
Platelet/Lymphocyte Ratio (PLR)	202.04 ± 298.20	157.95 ± 141.42	0.947
Neutrophil/Platelet Ratio (NPR)	0.094 ± 0.200	0.064 ± 0.055	0.129
HALP Score	26.49 ± 46.02	25.56 ± 21.65	0.072
CRP/Albumin Ratio	9.02 ± 17.93	6.28 ± 6.38	0.024
Clinical Severity Scores			
Glasgow Coma Scale (GCS)	7.85 ± 3.74	10.70 ± 3.66	<0.001
SOFA Score	8.21 ± 4.08	5.23 ± 3.03	<0.001
APACHE II Score	23.96 ± 9.02	14.03 ± 7.43	<0.001

NLR: Neutrophil/Lymphocyte Ratio, PLR: Platelet/Lymphocyte Ratio, NPR: Neutrophil/Platelet Ratio, CRP: C-reactive protein, SD: Standard deviation.

**Table 2:** Comparison of inflammatory indices and scoring systems according to mortality status in the malignancy subgroup.

Parameter	Non-Survivors (*n* = 30) Mean ± SD	Survivors (*n* = 14) Mean ± SD	*p*-Value
APACHE II Score	23.63 ± 7.42	15.28 ± 8.97	0.002
SOFA Score	7.33 ± 4.57	5.50 ± 2.90	0.329
Neutrophil/Lymphocyte Ratio (NLR)	12.64 ± 8.34	6.50 ± 4.15	0.008
Platelet/Lymphocyte Ratio (PLR)	137.79 ± 215.65	273.89 ± 185.24	0.003
Neutrophil/Platelet Ratio (NPR)	0.095 ± 0.096	0.061 ± 0.041	0.338
HALP Score	35.12 ± 34.66	14.10 ± 13.27	0.001
CRP/Albumin Ratio	10.23 ± 6.39	7.22 ± 5.95	0.047

NLR: Neutrophil/Lymphocyte Ratio. PLR: Platelet/Lymphocyte Ratio. NPR: Neutrophil/Platelet Ratio. CRP: C-reactive protein. SD: Standard deviation.

**Table 3:** Comparison of inflammatory indices and scoring systems according to mortality status in the sepsis subgroup.

Parameter	Non-Survivors (*n* = 100) Mean ± SD	Survivors (*n* = 34) Mean ± SD	*p*-Value
APACHE II Score	24.27 ± 9.48	14.17 ± 8.02	<0.001 *
SOFA Score	8.23 ± 4.07	5.61 ± 3.41	0.001 *
Neutrophil/Lymphocyte Ratio (NLR)	9.82 ± 7.22	8.17 ± 5.79	0.348
Platelet/Lymphocyte Ratio (PLR)	188.68 ± 206.24	232.34 ± 170.54	0.036 *
Neutrophil/Platelet Ratio (NPR)	0.098 ± 0.248	0.056 ± 0.053	0.130
HALP Score	28.50 ± 54.01	19.67 ± 18.17	0.638
CRP/Albumin Ratio	8.38 ± 5.92	8.79 ± 6.37	0.874

* Statistically significant at *p* < 0.05.

**Table 4:** Comparison of inflammatory indices and scoring systems according to mortality status in the trauma subgroup.

Parameter	Non-Survivors (*n* = 100) Mean ± SD	Survivors (*n* = 34) Mean ± SD	*p*-Value
APACHE II Score	25.71 ± 7.99	13.50 ± 6.14	<0.001 *
SOFA Score	10.57 ± 3.58	4.86 ± 2.82	<0.001 *
Neutrophil/Lymphocyte Ratio (NLR)	6.23 ± 3.93	4.07 ± 9.01	0.540
Platelet/Lymphocyte Ratio (PLR)	86.30 ± 62.94	96.21 ± 93.96	0.260
Neutrophil/Platelet Ratio (NPR)	0.094 ± 0.061	0.069 ± 0.050	0.140
HALP Score	29.83 ± 19.29	29.94 ± 19.29	0.920
CRP/Albumin Ratio	4.83 ± 6.85	4.13 ± 6.41	0.385

* Statistically significant at *p* < 0.05.

## Data Availability

Data are available from the corresponding author upon reasonable request.
